# Altered Treg Infiltration after Discoidin Domain Receptor 1 (DDR1) Inhibition and Knockout Promotes Tumor Growth in Lung Adenocarcinoma

**DOI:** 10.3390/cancers15245767

**Published:** 2023-12-08

**Authors:** Kathrin Maitz, Paulina Valadez-Cosmes, Sofia Raftopoulou, Oliver Kindler, Melanie Kienzl, Hamid Bolouri, A. McGarry Houghton, Rudolf Schicho, Akos Heinemann, Julia Kargl

**Affiliations:** 1 Division of Pharmacology, Otto Loewi Research Center, Medical University of Graz, 8010 Graz, Austria; 2 Center for Systems Immunology, Benaroya Research Center, Seattle, WA 98101, USA; 3 Human Biology Division, Fred Hutchinson Cancer Research Center, Seattle, WA 98109, USA; 4 Clinical Research Division, Fred Hutchinson Cancer Research Center, Seattle, WA 98109, USA; 5 Division of Pulmonary and Critical Care Medicine, University of Washington, Seattle, WA 98195, USA; 6 BioTechMed, 8010 Graz, Austria

**Keywords:** non-small cell lung cancer, discoidin domain receptor 1, tumor microenvironment, regulatory T cells

## Abstract

**Simple Summary:**

In Europe, seven out of eight patients with lung cancer die within 5 years after diagnosis. DDR1, a tyrosine kinase receptor, has emerged as a potential new therapeutic target for non-small cell lung cancer given its association with poor prognosis among affected patients. This study investigates the impact of DDR1 on tumor burden and immune cell infiltration into the tumor microenvironment. We found that pharmacological inhibition and knockout of DDR1 increased the tumor burden in an immunocompetent mouse model of lung adenocarcinoma. The absence of DDR1 reduced CD8^+^ cytotoxic T-cell infiltration but increased CD4^+^ helper and regulatory T-cell infiltration. Regulatory T cells, which promote tumorigenesis by suppressing the immune system, were also more common among The Cancer Genome Atlas (TCGA) lung adenocarcinoma patients with low DDR1 expression. These findings suggest that therapeutic inhibition of DDR1, in certain circumstances, might even have negative effects, although further studies are needed to confirm these findings.

**Abstract:**

Lung cancer is the leading cause of cancer-related death worldwide. Discoidin domain receptor 1 (DDR1), a tyrosine kinase receptor, has been associated with poor prognosis in patients with non-small cell lung cancer (NSCLC). However, its role in tumorigenesis remains poorly understood. This work aimed to explore the impact of DDR1 expression on immune cell infiltration in lung adenocarcinoma. Pharmacological inhibition and knockout of DDR1 were used in an immunocompetent mouse model of KRAS/p53-driven lung adenocarcinoma (LUAD). Tumor cells were engrafted subcutaneously, after which tumors were harvested for investigation of immune cell composition via flow cytometry. The Cancer Genome Atlas (TCGA) cohort was used to perform gene expression analysis of 509 patients with LUAD. Pharmacological inhibition and knockout of DDR1 increased the tumor burden, with DDR1 knockout tumors showing a decrease in CD8^+^ cytotoxic T cells and an increase in CD4^+^ helper T cells and regulatory T cells. TCGA analysis revealed that low-DDR1-expressing tumors showed higher FoxP3 (regulatory T-cell marker) expression than high-DDR1-expressing tumors. Our study showed that under certain conditions, the inhibition of DDR1, a potential therapeutic target in cancer treatment, might have negative effects, such as inducing a pro-tumorigenic tumor microenvironment. As such, further investigations are necessary.

## 1. Introduction

Among the 2.2 million new cancer cases worldwide in 2020, those diagnosed with lung cancer were the second most common, surpassed only by those diagnosed with breast cancer [1]. In Europe, lung cancer has a 5-year survival rate of only 12.6% [2], suggesting that lung cancer patients have yet to benefit from the advances in cancer diagnostics and treatment.

The tumor microenvironment (TME) contains not only cancer cells but also several other infiltrated non-malignant cells like stromal cells or immune cells, which all play a certain role in tumorigenesis. In non-small cell lung cancer (NSCLC), CD45^+^ leukocytes account for over 50% of all viable cells present within the tumor [3]. However, these leukocytes have been found to exhibit not only anti-tumorigenic but also pro-tumorigenic phenotypes. To evade the immune system, tumors create an immunosuppressive microenvironment by attracting immunosuppressive immune cells like regulatory T cells (Tregs), which can impact treatment outcomes, especially immunotherapy. Tregs, which are usually known for maintaining immune homeostasis by suppressing the immune system’s self-reactive immune responses, are the main tumor-promoting CD4^+^ helper T-cell subpopulation [4], and are associated with poor clinical prognosis in patients with NSCLC [5].

Discoidin domain receptor 1 (DDR1), a tyrosine kinase receptor, has been found to function as a sensor for the extracellular matrix (ECM) by regulating several cell functions, such as migration, adhesion, proliferation, cytokine secretion, and ECM homeostasis/remodeling. DDR is mostly expressed in epithelial cells, and its phosphorylation via collagen binding leads to the activation of various signaling pathways such as MAPK, integrin or Notch (reviewed in [6,7,8]). Nonetheless, several studies have suggested that DDR1 also exhibits collagen-independent activities [9]. In T cells, DDR1 expression has also been reported [10,11]; however, this expression is much lower compared to other cell types.

Studies show that DDR1 is overexpressed in several malignancies, such as lung [12], breast [13], brain [14], and gynecological cancers [15]. Over the last couple of years, several studies have shown an association between DDR1 and poor prognosis in patients with NSCLC [5,16], highlighting the potential of DDR1 as a new therapeutic target. Recently, multiple studies on breast [17,18,19] and colorectal cancers [20] have shown initial evidence that DDR1 could affect T-cell infiltration into the TME, mostly due to its interaction with collagen. To date, however, no link between DDR1 and T-cell infiltration in NSCLC has been established.

To investigate the role of DDR1 in promoting T-cell abundance in NSCLC, we used an immunocompetent mouse model of KRAS/p53-mutated lung adenocarcinoma (LUAD). Pharmacological inhibition and knockout of DDR1 increased the tumor burden and altered the T-cell composition. Increased CD4^+^ and Tregs and decreased CD8^+^ T-cell infiltration into DDR1-knockout tumors were observed. The Cancer Genome Atlas (TCGA) analysis of LUAD patients revealed that DDR1^low^ samples showed higher Forkhead box protein 3 (FoxP3, a Treg marker) expression than DDR1^high^ samples.

## 2. Materials and Methods

### 2.1. Cell Culture

The murine KP cell line (generously provided by Dr. A. McGarry Houghton, FredHutch, Seattle, WA, USA) was isolated from LUAD obtained from a Kras^LSL-G12D^ Trp53^Fl/Fl^ mouse with a C57BL/6 background after intratracheal administration of adenoviral Cre recombinase. Cells were cultured and grown in Dulbecco’s modified Eagle medium (DMEM) (Life Technologies, Vienna, Austria) with 10% fetal bovine serum (FBS) (Life Technologies, Vienna, Austria) and 1% penicillin/streptomycin (PS; PAN-Biotech, Aidenbach, Germany) in an incubator at 37 °C and 5% CO_2_. DDR1 knockout cell lines were selected using 10 µg/mL puromycin (ThermoFisher Scientific, Waltham, MA, USA).

### 2.2. DDR1-Knockout KP Cell Lines Using the CRISPR/Cas9 Lentivirus System

DDR1 oligos (Supplementary Table S1) (Eurofins, Louisville, KY, USA) were first cloned into the lentiCRISPR v2 (#52961; Addgene, Watertown, MA, USA) according to the “Target Guide Sequence Cloning Protocol” [21,22]. The plasmids were then transformed into Stbl3 bacteria (ThermoFisher Scientific, Waltham, MA, USA) with heat shock for 90 s at 42 °C. Bacteria were then cultured on agar plates with ampicillin (100 µg/mL; Sigma-Aldrich, St., Saint Louis, MO, USA) overnight at 37 °C. The next day, single clones were picked and cultured in 5 mL of LB medium (Sigma-Aldrich, St., Saint Louis, MO, USA) with 100 µg/mL ampicillin at 37 °C for 8 h with shaking. Thereafter, 1 mL of bacteria preculture was inoculated into 10 mL of LB medium with 100 µg/mL ampicillin and incubated overnight at 37 °C with shaking. On the next day, plasmids were isolated using the QIAGEN Plasmid Plus Mini Kit (Qiagen, Venlo, The Netherlands) and sequenced for right ligation (Eurofins, Louisville, KY, USA).

Lentivirus was produced using Lipofectamine™ 3000 (ThermoFisher Scientific, Waltham, MA, USA) in HEK293T cells according to the manufacturer’s instructions. The CRISPR plasmids described earlier were transfected together with packaging and envelope plasmids psPAX2 (#12260; Addgene, Watertown, MA, USA) and pMD2.G (#12259; Addgene, Watertown, MA, USA), respectively. HEK293T cells were incubated for 4 h with the DNA–lipid complex before the medium was changed. After 48 and 72 h, the supernatant containing the lentivirus was collected. KP cells were then seeded into a 6-well plate and incubated with the lentivirus for 48 h. Afterwards, cells were recovered for 48 h in DMEM + 10% FBS + 1% PS followed by selection with 20 µg/mL puromycin (ThermoFisher Scientific, Waltham, MA, USA). Single-cell clones were then selected and grown by seeding 1 cell into a 96-well plate, with each well containing 200 µL of the medium. After expansion, knockout clones were validated via Western blotting and the quantitative polymerase chain reaction (qPCR).

### 2.3. Murine Tumor Models

Age-matched male C57BL/6J mice, purchased from Charles River, Germany, were used for in vivo experiments. Experiments were approved by the Austrian Federal Ministry of Science and Research (BMBWF-66.010/0041-V/3b/2018). For the pharmacological inhibition of DDR1, 0.5 × 10^6^ KP cells were injected, and mice were treated daily with either 7rh inhibitor (Sigma-Aldrich, St., Saint Louis, MO, USA) or the vehicle intraperitoneally at a dosage of 8 mg/kg/day. After 14 days, mice were sacrificed, and tumors were harvested. For DDR1 knockout experiments, 0.325 × 10^6^ KP DDR1 knockout KO2 and KO6 or control (endogenous DDR1) were subcutaneously injected into the flank of mice. After 19 days, mice were sacrificed, and tumors were harvested. Tumors were weighed and measured for size. Tumor volume was calculated using the following formula: v = length × width × height × *π*/6. Tumor pieces were frozen for protein and RNA analysis or were used to create single-cell suspensions for flow cytometry.

### 2.4. Tumor Single-Cell Suspension

Tumors were minced into small pieces and transferred into 0.5–1 mL of a digestion medium (RPMI + 40 U/mL DNase I and 150 U/mL collagenase type 1 (both from Worthington Biochemical Corporation, Lakewood, NJ, USA)). Digestion was performed for a total of 25 min on a thermoshaker (37 °C with shaking). After 10 min, tissues were triturated with a pipette. Homogenization was performed via passage through a 16-gauge needle followed by a 40 µm cell strainer (Greiner, Kremsmünster, Austria) and by washing with SB (staining buffer: phosphate-buffered saline (PBS) + 2% FBS). After washing with PBS, the cells were resuspended and counted on an EVE automated cell counter (NanoEntek, Seoul, Republic of Korea), and 2 × 10^6^ cells were used for flow cytometry staining.

### 2.5. Flow Cytometry

Tumor single-cell suspensions were stained with Fixable Viability Dye eFluor™ 780 (eBioscience, ThermoFisher Scientific, Waltham, MA, USA) for 20 min at 4 °C. After washing with SB (staining buffer: PBS + 2% FBS), cells were incubated with surface antigen antibodies for 20 min after 10 min of blocking with TruStain FcX™ antibody (BioLegend, San Diego, CA, USA) at 4 °C. Fixation and permeabilization were performed using the Transcription Factor Buffer Set (BD Bioscience, Franklin Lakes, NJ, USA) for 40 min at 4 °C. Blocking was repeated, and nuclear antibodies were stained for 20 min at 4 °C. After washing with SB, the samples were measured using a BD LSR Fortessa (BD Bioscience, Franklin Lakes, NJ, USA) flow cytometer and analyzed using FlowJo™ software (v10.9.0, BD Life Science, Ashland, OR, USA). The following antibodies were used: CD45-AF700, CD8-PerCPCy5.5, NKp46-BV510, CD19-FITC, CD62L-BV605, PD-1-APC (all from BioLegend, San Diego, CA, USA), CD3-BUV395, CD4-BUV496, CD44-BUV737 (all from BD Bioscience, Franklin Lakes, NJ, USA) and FoxP3-PE (eBioscience, ThermoFisher Scientific, Waltham, MA, USA).

### 2.6. Western Blotting

Cells were lysed using IP lysis buffer (0.1% Triton X, 150 mM of NaCl, 25 mM of KCl, 10 mM of CaCl_2_) supplemented with protease/phosphatase inhibitor cocktail (Cell Signaling Technology, Danvers, MA, USA). Frozen tumor pieces were homogenized using a Precelly homogenizer (VWR, Radnor, PA, USA) and 1.4 mm ceramic beads (VWR, Radnor, PA, USA) in the same lysis buffer mentioned previously. Protein concentrations were adjusted using the Pierce™ BCA Protein Assay (ThermoFisher Scientific, Waltham, MA, USA). Denaturation was conducted using NuPAGE^TM^ Reducing Agent (10×) (ThermoFisher Scientific, Waltham, MA, USA) and NuPAGE™ LDS Sample Buffer (4×) (ThermoFisher Scientific, Waltham, MA, USA) for 10 min at 95 °C. After separation on a NuPAGE™ 4%–12% Bis-Tris protein gel (ThermoFisher Scientific, Waltham, MA, USA) and transfer to a polyvinylidene fluoride membrane, blocking was performed using 5% BSA (Sigma-Aldrich, St., Saint Louis, MO, USA) in TBST buffer (25 mM of Tris base, 135 mM of NaCl, 2.5 mM of KCl, 1 mM of CaCl_2_, 0.1% Tween 20, pH = 7.4) for 30 min. Incubation with the primary antibody (1:1000 DDR1, D1G6 rabbit mAb; 1:5000 GAPDH, 14C10 rabbit mAb; Cell Signaling Technology, Danvers, MA, USA) was performed overnight at 4 °C. Thereafter, incubation with the secondary antibody (1:10,000 goat anti-rabbit HRP antibody, Jackson ImmunoResearch, Philadelphia, PA, USA) was performed for 1.5 h at room temperature. Membranes were developed using Clarity Western ECL substrate (BioRad, Hercules, CA, USA) on a ChemiDoc™ Touch Imaging System (BioRad, Hercules, CA, USA).

### 2.7. RNA Extraction and qPCR

RNA extraction was performed using the RNeasy Mini Kit (Qiagen, Venlo, The Netherlands) according to the manufacturer’s protocol. Tumor pieces were previously homogenized using a Precelly homogenizer (VWR, Radnor, PA, USA) and 1.4 mm ceramic beads (VWR, Radnor, PA, USA). Reverse transcription was performed using the High-Capacity cDNA Reverse Transcription Kit (ThermoFisher Scientific, Waltham, MA, USA) according to the manufacturer’s protocol on a Thermal Cycler (BioRad, Hercules, CA, USA). Real-time PCR was performed using TaqMan Gene Expression Master Mix (ThermoFisher Scientific, Waltham, MA, USA) according to the manufacturer’s protocol and probes for DDR1 (Mm01273496_m1) and GAPDH (Mm99999915_g1) (ThermoFisher Scientific, Waltham, MA, USA) on a CFX Connect Real-Time System (BioRad, Hercules, CA, USA).

### 2.8. BrdU Proliferation Assay

Cells were seeded in a 6-well plate and grown for 24 h. Thereafter, 10 µM of BrdU was added and incubated for 1 h at 37 °C. Cells were detached and stained with BrdU-FITC antibody as described in the manufacturer’s protocol (FITC BrdU Flow Kit, BD Bioscience, Franklin Lakes, NJ, USA).

### 2.9. Analysis of TCGA DDR1 Expression Data

Gene expression and mutation data were obtained from the GDC TCGA cohort, downloaded via the Xena Browser (https://xenabrowser.net/, accessed on 7 February 2022). The gene expression data (HTSeq-FPKM-UQ) comprised a cohort of 877 clinical LUAD samples, with 597 primary tumor data from 509 patients. Subsequently, one sample per patient was randomly selected, and these samples were categorized based on DDR1 expression (high vs. low). The selected genes were then compared between these groups using an unpaired Student’s *t*-test. Furthermore, patients were grouped based on their mutation status of selected genes, and DDR1 expression was compared using an unpaired Student’s *t*-test. The analysis was performed using R software (v4.1.2).

### 2.10. Statistical Analysis

Statistical analysis was performed using GraphPad Prism 9 (GraphPad Software, La Jolla, CA, USA). Data were tested for Gaussian distribution using the Shapiro–Wilk test of normality. Significant outliers were calculated using the GraphPad Outlier calculator (Grubb’s test) and excluded from statistical analysis. For parametric data, Student’s *t*-test with Welch correction was performed using data from the two groups. Ordinary one-way ANOVA with Dunnett’s multiple comparison test was used to analyze data of three different groups. For non-parametric data, the Mann–Whitney test or Kruskal–Wallis test with Dunn’s multiple comparisons test was performed. Results are presented as mean + SD. A *p* value of <0.05 indicated statistical significance.

## 3. Results

### 3.1. Inhibition and Knockout of DDR1 Drives Tumor Growth in Mice

**Figure 1:**
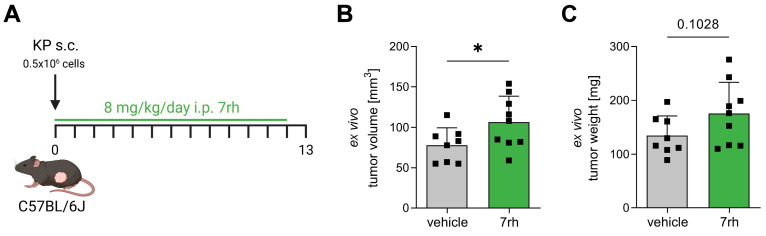
Pharmacological inhibition of DDR1 drives tumor growth in an in vivo model of lung adenocarcinoma. (**A**) Experimental protocol of the in vivo syngeneic mouse model. KP lung adenocarcinoma cells were injected subcutaneously (s.c.) into the flank of C57BL/6J wild-type mice on day 0. Furthermore, mice were treated with 8 mg/kg of the DDR1 inhibitor 7rh or the vehicle via daily intraperitoneal (i.p.) injections. On day 13, mice were sacrificed, and tumors were harvested. (**B**,**C**) Ex vivo measured tumor volume (**B**) and weight (**C**) (*n* = 8 to 9). * *p* < 0.05 using Student’s *t*-test. Data are presented as mean + SD.

Recent studies on in vivo models of breast and colorectal cancers have shown that DDR1 affects immune cell composition and infiltration into the TME [17,18,19,20]. To date, however, it remains unknown whether DDR1 also impacts T-cell infiltration in NSCLC. To address this, we used an in vivo model of immunocompetent LUAD and pharmacologically inhibited DDR1 (Figure 1). The TCGA lung cancer patient dataset revealed an upregulation of DDR1 in patients with a KRAS or EGFR mutation, but not in those with STK11/LKB1 or TP53 mutations (Supplementary Figure S1). Due to the absence of an available EGFR mutant mouse model, we chose to utilize a KRAS/p53-driven model. Accordingly, male C57BL/6J mice were subcutaneously injected with the KP cell line (isolated from LUAD derived from a Kras^LSL-G12D^ Trp53^Fl/Fl^ mouse with a C57BL/6 background [23,24]) and were treated with 8 mg/kg of the DDR1 inhibitor 7rh or the vehicle via daily intraperitoneal injections (Figure 1A). After 13 days, inhibitor-treated mice surprisingly showed increased ex vivo tumor volume (Figure 1B) and weight (Figure 1C).

To further investigate the results from the pharmacological inhibition of DDR1, we next created DDR1-knockout KP cell lines using the CRISPR/Cas9 lentivirus system. Different guide RNA sequences (Supplementary Table S1) were used to create KO cell lines. Knockout was validated using Western blotting (Supplementary Figure S2A,B) and qPCR (Supplementary Figure S2C). We also assessed whether the knockout altered proliferation in vitro using a bromodeoxyuridine (BrdU) flow cytometry assay. However, the different cell lines showed no difference in proliferation in vitro compared to the parental KP cell line (Supplementary Figure S2D).

**Figure 2:**
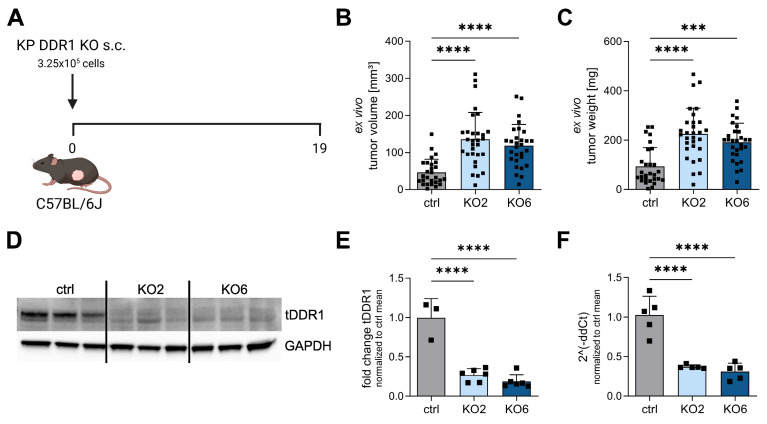
DDR1 knockout increases tumor growth in vivo. (**A**) Schematic representation of the experimental procedure performed on the in vivo syngeneic mouse model. C57BL/6J wild-type mice were injected subcutaneously (s.c.) with KP DDR1 knockout (KO2 and KO6) and control (ctrl) cells. After 19 days, mice were sacrificed, and tumors were harvested. Three independent experiments were then performed. (**B**,**C**) Ex vivo tumor volume (**B**) and weight (**C**) were measured at the end of the experiment. (*n* = 28–30). (**D**,**E**) Western blotting showing the DDR1 expression of lysed tumor tissues. GAPDH was used as loading control. Original Western blots and intensity ratios are shown in Supplementary Figure S3. (**F**) DDR1 RNA levels of lysed tumor tissues. Samples were normalized to GAPDH. *** *p* < 0.0005, **** *p* < 0.0001 using one-way ANOVA. Data shown are mean + SD.

Based on knockout validation, we injected KO2, KO6, and the control (ctrl, endogenous DDR1) subcutaneously into the flank of male C57BL/6J mice (Figure 2A). After 19 days, DDR1-knockout tumors showed a significant increase in ex vivo volume (Figure 2B) and weight (Figure 2C) compared to the control, which supports the inhibitor data.

To test whether DDR1 knockout is stable in vivo and whether DDR1 is mainly expressed by the cancer cells, Western blotting and qPCR were performed on homogenized frozen tumor tissues. GAPDH was used as the control housekeeping gene in both protein and RNA analyses. DDR1-knockout tumors showed decreased DDR1 expression in vivo at the protein (Figure 2D,E) and RNA levels (Figure 2F).

### 3.2. DDR1 Knockout Leads to a Pro-Tumorigenic T-Cell Profile In Vivo

**Figure 3:**
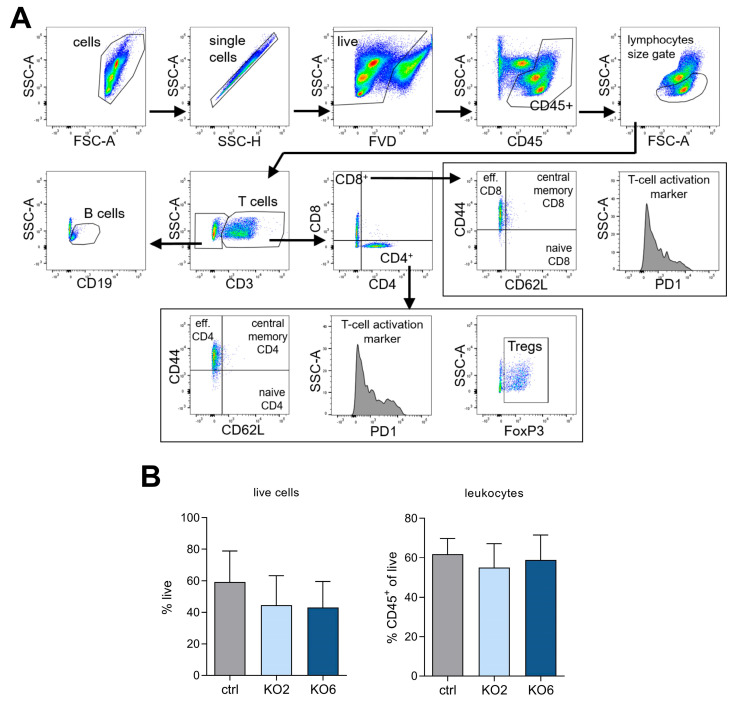
Immune cell composition in DDR1-knockout tumors. (**A**) Representative dot blots of the gating strategy used for immune cell flow cytometry panel in in vivo experiments. Cells were gated for singlets to exclude doublets. Single cells were then gated for viability (FVD) and leucocyte marker (CD45^+^). Leukocytes were gated based on lymphocyte size followed by a T-cell (CD3^+^) gate. B (CD19^+^) cells were gated for non-T cells (CD3^−^). T cells were gated for CD4 helper (CD4^+^) and CD8 cytotoxic (CD8^+^) T cells. CD4 and CD8 T cells were subsequently gated for naive T cells (CD44^−^, CD62L^+^), central memory T cells (CD44^+^, CD62L^+^), effector T cells (CD44^+^, CD62L^−^), and T-cell activation marker (PD-1). CD4^+^ T cells were additionally gated for regulatory T cells (Tregs, FoxP3^+^). FMOs of CD45, CD62L and CD44 are shown in Supplementary Figure S4. (**B**) Immune cell flow panel data for ctrl, KO2, and KO6 single-cell suspensions. Shown are %live cells and %CD45^+^ leukocytes (*n* = 14–15). Data regarding T-cell subpopulations are shown in Figure 4 and Figure 5. Statistical analysis was performed using one-way ANOVA. Data are presented as mean + SD.

To investigate whether DDR1 knockout altered immune cell infiltration into the TME, we created tumor single-cell suspensions and performed staining for a lymphoid flow cytometry panel (Supplementary Table S2). The representative gating strategy is shown in Figure 3A. DDR1-knockout tumors show no changes in live cells (% of singlets) or infiltrated leukocytes (%CD45^+^ of live) (Figure 3B).

**Figure 4:**
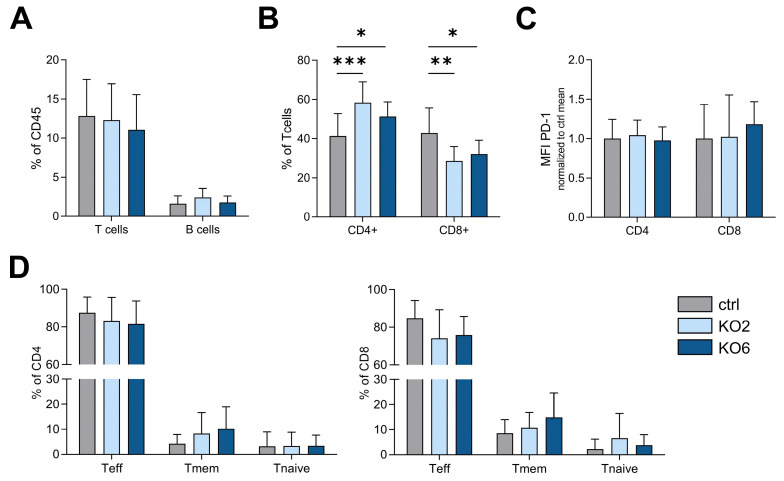
DDR1 knockout in the KP lung adenocarcinoma model causes altered T-cell composition in vivo. Flow cytometry analysis of tumor single-cell suspensions pooled from two independent experiments (*n* = 14–15). The gating strategy is shown in Figure 3. (**A**) CD3^+^ T cells and CD19^+^ B cells according to the percentage of CD45^+^ leukocytes. (**B**) CD4^+^ helper T cells and CD8^+^ cytotoxic T cells according to percentage of CD3^+^ T cells (**C**) PD-1 median fluorescence intensity (MFI) of CD4^+^ and CD8^+^ T cells. (**D**) Effector (CD44^+^, CD62L^−^), central memory (CD44^+^, CD62L^+^), and naive (CD44^−^, CD62L^+^) T cells are shown according to the percentage of CD4^+^ and CD8^+^ T cells. * *p* < 0.05, ** *p* < 0.005, *** *p* < 0.0005 using one-way ANOVA. Data are presented as mean + SD.

Furthermore, no changes were observed in total T- or B-cell infiltration (Figure 4A). Regarding T-cell (CD3^+^) composition, however, differences in CD4^+^ helper T-cell and CD8^+^ cytotoxic T-cell abundance were observed among tumors. Both KO2 and KO6 tumors showed an increase in CD4^+^ helper T cells but a decrease in CD8^+^ T cells compared to the control (Figure 4B).

Moreover, no changes were observed in the median fluorescence intensity of inhibitory checkpoint receptor PD-1, which indicates T-cell activation status (Figure 4C), or in effector, memory, or naive T-cell distribution of CD4^+^ helper T cells or CD8^+^ cytotoxic T cells (Figure 4D).

**Figure 5:**
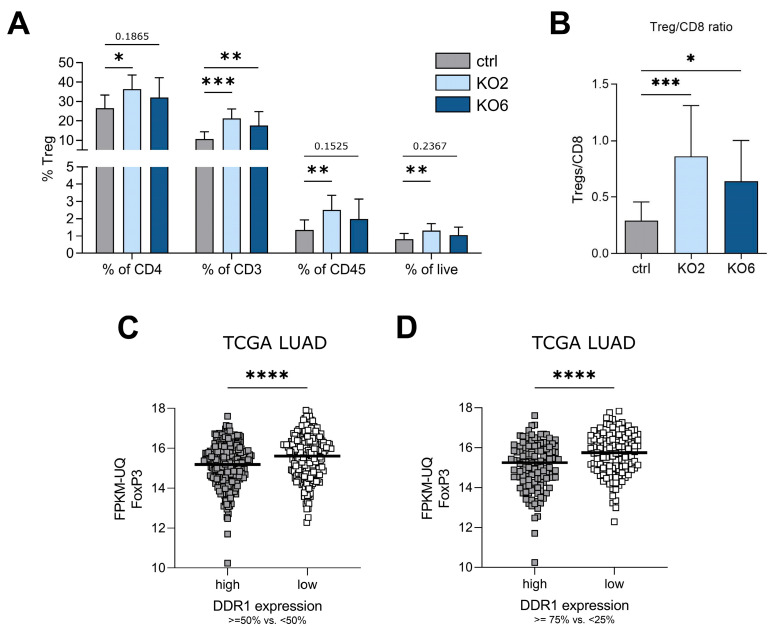
Low DDR1 expression increases regulatory T-cell abundance in vivo and in TCGA human lung adenocarcinoma data. (**A**,**B**) Flow cytometry analysis of tumor single-cell suspensions pooled from two independent experiments (*n* = 14–15). The gating strategy is shown in Figure 3. (**A**) In vivo knockout increased regulatory T-cell (Tregs) infiltration. Regulatory T cells are shown according to the percentage of CD4^+^, CD3^+^, CD45^+^, and live cells. (**B**) Tregs/CD8 ratio in tumors in vivo. * *p* < 0.05, ** *p* < 0.005, *** *p* < 0.0005 using one-way ANOVA. Data are presented as mean + SD. (**C**,**D**) Gene expression of FoxP3 (a regulatory T-cell marker) in lung adenocarcinoma (LUAD) patient samples with high/low DDR1 expression. Expression data were provided through The Cancer Genome Atlas (GDC TCGA) and included 877 clinical samples, 597 primary tumor data, and 509 patients. (**C**) Low/high DDR1 expression was categorized as <50% vs. ≥50% and (**D**) <25% vs. ≥75%, respectively. **** *p* < 0.0001 using the Mann–Whitney test. TCGA LUAD, The Cancer Genome Atlas Lung Adenocarcinoma; FPKM-UQ, fragments per kilobase of transcript per million mapped reads upper quartile.

Within the CD4^+^ helper T-cell population, we found that Treg infiltration was increased in the DDR1-knockout tumors (Figure 5A). FoxP3, which is a Treg transcription factor, was elevated in CD4^+^ helper T cells, CD3^+^ T cells, CD45^+^ leukocytes, and live cells. An investigation into the Treg-to-CD8 ratio showed a significant increase in the ratio among DDR1-knockout tumors (Figure 5B).

### 3.3. Low DDR1 Expression in LUAD Patients Shows Higher FoxP3^+^ Treg Expression

Given that knockout tumors showed an increase in Tregs, we next investigated a gene expression patient dataset of LUAD samples from TCGA. A cohort of 877 clinical samples with 597 primary tumor data from 509 patients were analyzed. Samples were grouped according to DDR1 expression (low, <50% vs. high, ≥50% or low, <25% vs. high, ≥75%) and were compared to FoxP3, a nuclear transcription factor present in Tregs that represents the main tumor-promoting CD4^+^ T-cell population. Notably, the DDR1^low^ group showed higher FoxP3 expression than the DDR1^high^ group (Figure 5C,D). Given that we employed a KRAS/p53-driven mouse model, we also investigated FoxP3 expression in patients with a KRAS/p53 double mutation (Supplementary Figure S5), revealing a significant increase in the <50% vs. ≥50% group. However, it is essential to note that the sample numbers are relatively low.

## 4. Discussion

Over the last couple of years, T-cell-targeted immunotherapy has gained more and more attention in cancer treatment research. The infiltration of T cells into the TME is a crucial step in obtaining efficient immune response against cancer cells and is consequently important for the success of cancer therapy.

The tyrosine kinase receptor DDR1, which regulates various cell functions such as migration, adhesion, proliferation, cytokine secretion, and ECM homeostasis/remodeling (reviewed in [6,7]), has been discussed as a possible new target for cancer treatment given that it is not only overexpressed in lung cancer [12] but also associated with poor prognosis in patients with NSCLC [5,16]. Ambrogio et al. showed that the combined inhibition of DDR1 and Notch signaling decreased tumor growth in a mouse model of KRAS-driven LUAD [25]. Moreover, DDR1 inhibition enhanced the in vivo chemosensitivity of KRAS-mutant LUAD [26]. Unfortunately, no study has yet investigated whether DDR1 affects immune cell invasion into the TME of NSCLC. However, initial evidence from recent studies on breast [17,18,19] and colorectal cancers [20] have shown that DDR1 could play a role in T-cell infiltration. In fact, a study on breast cancer patients by Sun et al. revealed a negative correlation between DDR1 expression and CD8^+^ T cells in breast cancer [17]. Another study showed that targeting DDR1 with a humanized monoclonal antibody reversed immune exclusion by increasing T-cell infiltration and significantly increased antitumor efficacy in a mouse model of immunocompetent breast cancer [18]. Another breast cancer study recently revealed that collagen-induced DDR1 upregulated CXCL5, which promoted the formation of NETs and enhanced Treg infiltration, thereby facilitating the growth and metastasis of breast cancer [19]. Duan et al., who studied colorectal cancer, demonstrated that DDR1 promoted tumor growth in vivo by inhibiting IL-18 synthesis, leading to the decreased infiltration of CD4^+^ and CD8^+^ T cells [20]. The lack of studies investigating the potential involvement of DDR1 in immune cell infiltration in NSCLC sparked our interest on this matter.

Surprisingly, the current study showed that in an immunocompetent mouse model of KRAS/p53 LUAD, disturbing DDR1 increased the tumor burden. Both pharmacological inhibition (i.e., through DDR1 inhibitor 7rh) and genetic knockout of DDR1 promoted an increase in tumor volume in a KP mouse model. Our analysis of the infiltrated immune cells found no changes in leucocyte, general T-cell, or B-cell abundance; however, it did show differences in the presence of CD8^+^ and CD4^+^ T-cell subsets. In the DDR1-knockout tumors, we observed a decrease in CD8^+^ cytotoxic T cells and an increase in CD4^+^ helper and Tregs. The decrease in CD8^+^ cytotoxic T cells, which play a vital role in directly attacking and eliminating cancer cells, suggests that the absence of DDR1 might impact the recruitment or proliferation of CD8^+^ cytotoxic T cells, leading to reduced tumor cell killing.

The changes in Tregs with DDR1 knockout were also observed in the TCGA gene expression dataset on lung adenocarcinoma patients. We observed that the expression of FoxP3, a marker for Tregs, was increased in tumors with low DDR1 expression and decreased in those with high DDR1 expression. The increase in Tregs could have a pro-tumorigenic effect in tumor growth given that their presence can limit the effectiveness of antitumor immunity and contribute to immune evasion. These changes in T-cell subsets might hint at a disturbed balance between immune-activating and immunosuppressive T cells, leading to a pro-tumorigenic TME in this immunocompetent model.

Overall, our study highlights the potential for DDR1 to play an important role in modulating the composition and function of T cells within the TME of NSCLC.

## 5. Conclusions

The findings presented herein show that DDR1 might exert some anti-tumorigenic effects in immunogenic lung cancer models. However, further investigations are needed to assess the potential of DDR1 as a therapeutic target given that it might induce Treg infiltration and/or differentiation, causing immunosuppression in hot tumors.

## Data Availability

The authors declare that all the data supporting the findings of this study are available from the corresponding author upon reasonable request.

## Supplementary Materials

The following supporting information can be downloaded at: https://www.mdpi.com/article/10.3390/cancers15245767/s1. Supplementary Table S1: DDR1 oligos used for CRISPR/Cas9 knockout of DDR1; Supplementary Table S2: Antibodies used for flow cytometry staining of tumor single-cell suspensions; Supplementary Figure S1: DDR1 expression in lung adenocarcinoma (LUAD) patients with different mutations (MUT) compared to wildtype (WT). DDR1 expression is shown as FPKM-UQ (fragments per kilobase of transcript per million mapped reads upper quartile) using TCGA (The Cancer Genome Atlas) data.; Supplementary Figure S2: Validation of DDR1 knockout cell lines. (A) The Western blot shows the DDR1 expression of knockout KP cell lines in vitro. Ctrl was used as the control, whereas KO2 and KO6 were used as knockout cell lines. GAPDH was used as a loading control. Original Western blots and intensity ratios are shown in Supplementary Figure S6. (B) Western blots were normalized to ctrl within each experiment (*n* = 3). (C) In vitro DDR1 RNA levels in each cell line. (D) In vitro proliferation of DDR1 knockout cell lines using bromodeoxyuridine (BrdU) flow cytometry analysis. * *p* < 0.05, **** *p* < 0.0001 using one-way ANOVA. Data shown are mean + SD; Supplementary Figure S3: Intensity ratios and Western blots for Figure 2D,E. (A) Values indicate the intensity ratio of DDR1/GAPDH. Ctrl samples on Gel 1 and 2 are identical. Ctrl values in Figure 2E are the mean of each ctrl of both gels. (B) tDDR1 (125 kDa) and (C) GAPDH (37 kDa) Western blot gels; Supplementary Figure S4: Fluorescence minus one (FMO) of CD45, CD62L, and CD44 gating; Supplementary Figure S5: Gene expression of FoxP3 (a regulatory T-cell marker) in lung adenocarcinoma (LUAD) patient samples with a KRAS and p53 (KP) mutation with high/low DDR1 expression. Expression data were provided through The Cancer Genome Atlas (GDC TCGA). Low/high DDR1 expression was categorized as <50% vs. ≥50% (group50); Supplementary Figure S6: Intensity ratios and Western blots for Supplementary Figure S2A. (A) Values indicate the intensity ratio of DDR1/GAPDH. (B) tDDR1 (125 kDa) and (C) GAPDH (37 kDa) Western blot gels.
