# Supplementary material for: Altered Treg Infiltration after Discoidin Domain Receptor 1 (DDR1) Inhibition and Knockout Promotes Tumor Growth in Lung Adenocarcinoma
Source: Cancers (Basel). 2023 Dec 8;15(24):5767. doi: 10.3390/cancers15245767 (PMC10742023; doi:10.3390/cancers15245767)
Supplement: Supplementary file 1 [file cancers-15-05767-s001.zip › cancers-2627944-supplementary.pdf]

Supplementary

Altered Treg Infiltration after Discoidin Domain Receptor 1 (DDR1) Inhibition and Knockout Promotes Tumor Growth in Lung Adenocarcinoma

Table S1. DDR1 oligos used for CRISPR/Cas9 knockout of DDR1.

# of knockout	Oligo	Sequence
1	mDDR1_1_A for	CACCGCCTGACGCCCGGGTCCGCCC
	mDDR1_1_A rev	AAACGGGCGGACCCGGGCGTCAGGC
2	mDDR1_2_A for	CACCGGCCCAGGGCATAGCGGCACT
	mDDR1_2_A rev	AAACAGTGCCGCTATGCCCTGGGCC
4	mDDR1_4_A for	CACCGCTGTCATATACAGCCCCCGT
	mDDR1_4_A rev	AAACACGGGGGCTGTATATGACAGC
6	mDDR1_6_A for	CACCGACTGCAGAAAGCGGCCCACG
	mDDR1_6_A rev	AAACCGTGGGCCGCTTTCTGCAGTC

Table S2. Antibodies used for flow cytometry staining of tumor single cell suspensions.

Antibody	Fluorophor	Titer/Test	Company	cat#
FVD	APC-Cy7	1:2000	ThermoFisher Scientific	65-0865-18
CD45	AF700	0.25	Biolegend	103128
CD3	BUV395	1.25	BD	563565
CD4	BUV496	0.625	BD	612952
CD8	PerCP-Cy5.5	0.625	Biolegend	100734
CD19	FITC	0.31	Biolegend	115506
CD44	BUV737	0.31	BD	612799
CD62L	BV605	1	Biolegend	104438
PD-1	APC	1.25	Biolegend	135210
FoxP3	PE	1.25	eBio	12-5773–82

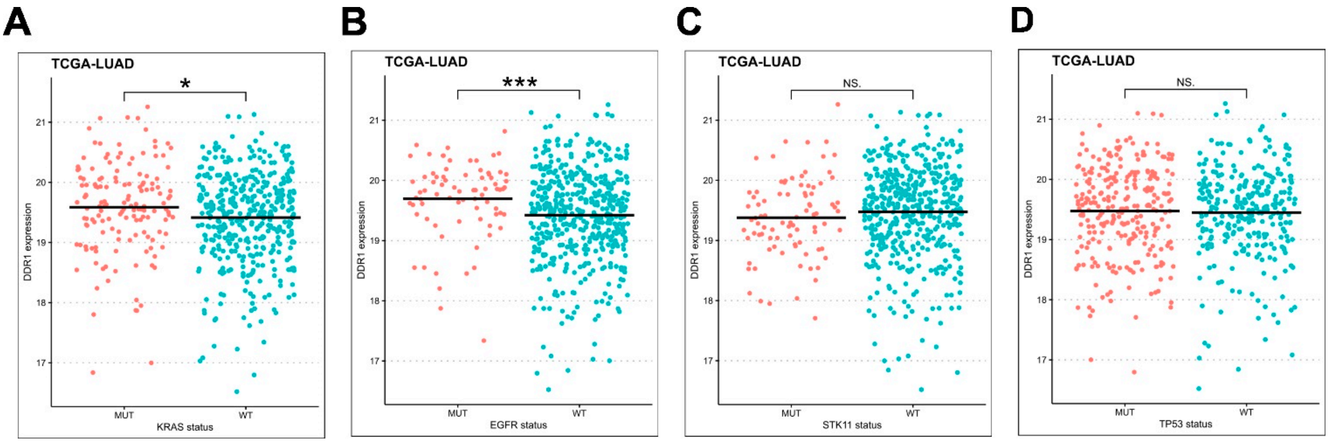

Figure S1. DDR1 expression in lung adenocarcinoma (LUAD) patients with different mutations (MUT) compared to wildtype (WT). Shown are (**A**) KRAS (**B**) EGFR (**C**) STK11 and (**D**) TP53 mutation status. DDR1 expression is shown as FPKM-UQ (Fragments Per Kilobase of transcript per Million mapped reads Upper Quartile) using TCGA (The Cancer Genome Atlas) data. **p* < 0.05, ****p* < 0.0005 using unpaired Student's t-test.

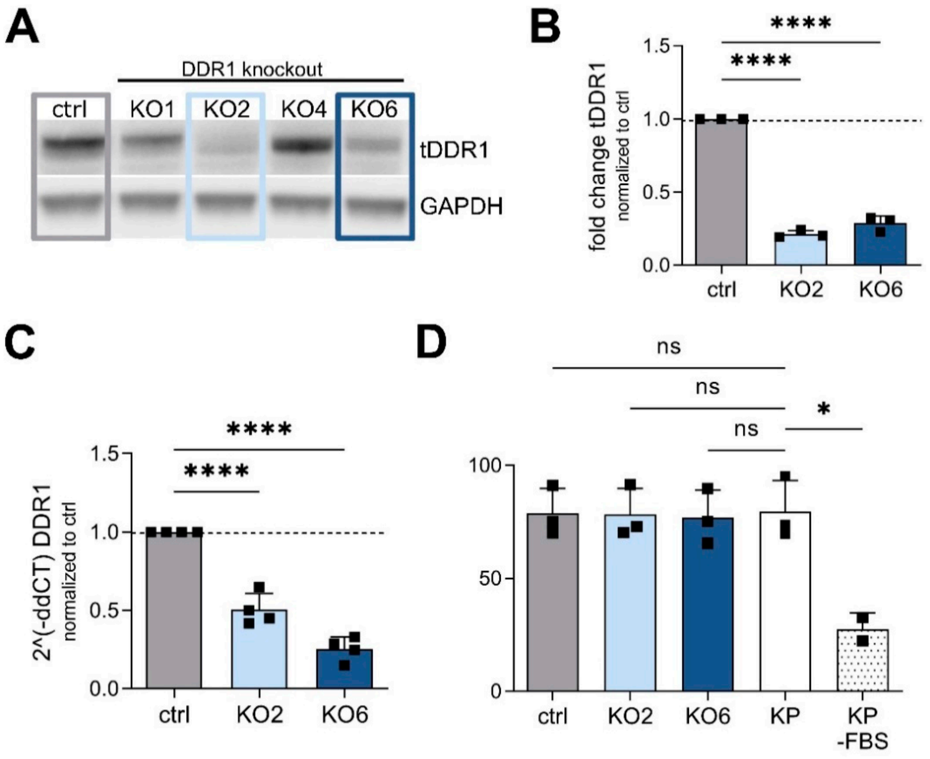

Figure S2. Validation of DDR1 knockout cell lines. (**A**) The Western blot shows the DDR1 expression of knockout KP cell lines in vitro. Ctrl was used as control, whereas KO2 and KO6 were used as knockout cell lines. GAPDH was used as a loading control. Original western blots and intensity ratios are shown in Supplementary Fig. 6. (**B**) Western blots were normalized to ctrl within each experiment (*n* = 3). (**C**) In vitro DDR1 RNA levels in each cell line. (**D**) In vitro proliferation of DDR1 knockout cell lines using bromodeoxyuridine (BrdU) flow cytometry analysis. **p* < 0.05, *****p* < 0.0001 using one-way ANOVA. Data shown are mean + SD.

A

	Lane 1	Lane 2	Lane 3	Lane 4	Lane 5	Lane 6	Lane 7	Lane 8	Lane 9
**Gel 1 DDR1/GAPDH**	1,144	1,141	0,699	0,365	0,281	0,329	0,165	0,126	0,359
**Gel2 DDR1/GAPDH**	1,079	1,182	0,726	0,171	0,291	0,170	0,155	0,183	0,134
**ctrl mean**	1,111	1,162	0,713						

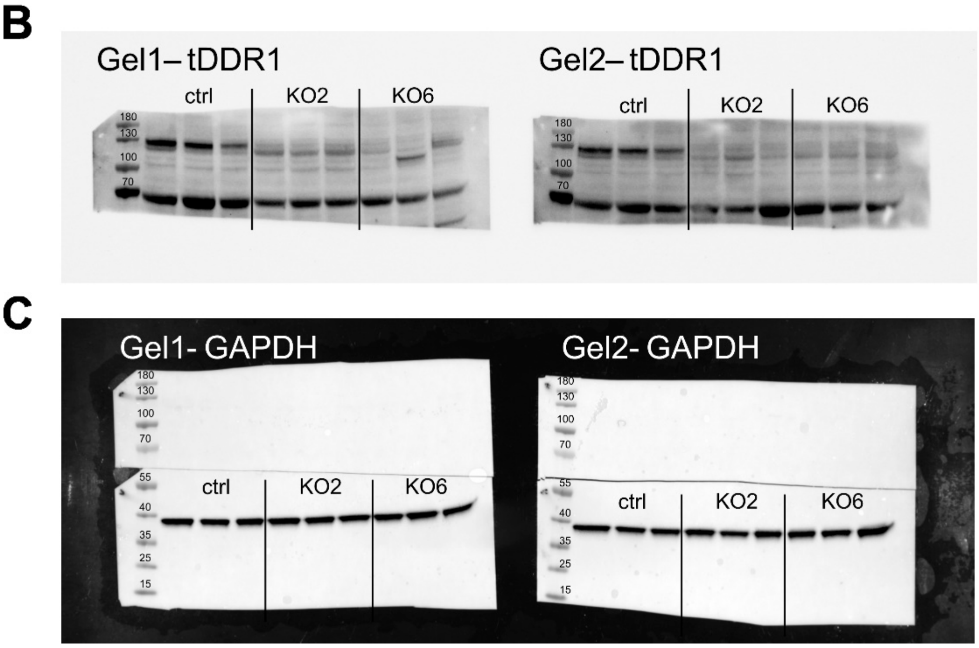

Figure S3. Intensity ratios and western blots for Figure 2 D and E. (**A**) Values indicate intensity ratio DDR1/GAPDH. Ctrl samples on Gel 1 and 2 are identical. Ctrl values in Figure 2E are the mean of each ctrl of both gels. (**B**) tDDR1 (125 kDa) and (**C**) GAPDH (37 kDa) western blot gels.

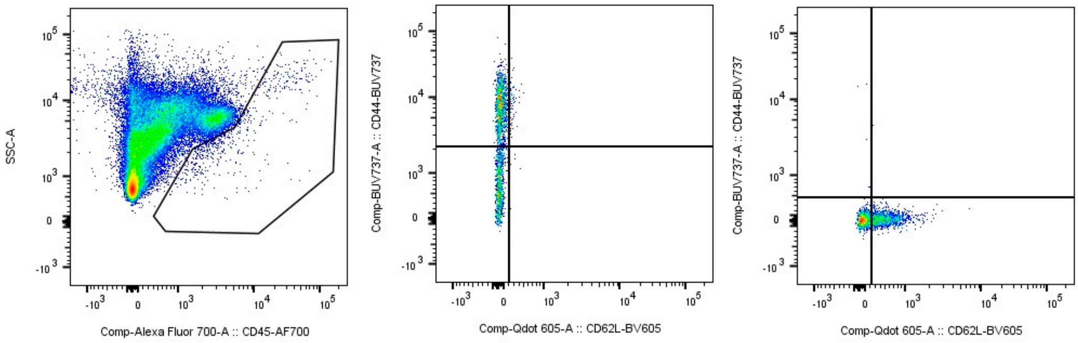

Figure S4. Fluorescence Minus One (FMO) of CD45, CD62L, and CD44 gating.

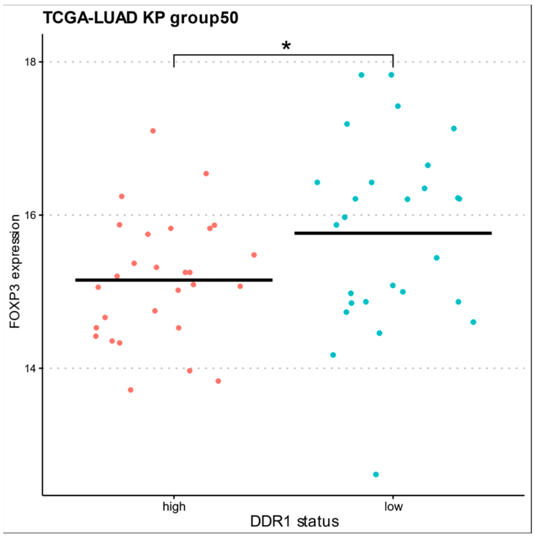

Figure S5. Gene expression of FoxP3 (a regulatory T cell marker) in lung adenocarcinoma (LUAD) patient samples with a KRAS and p53 (KP) mutation with high/low DDR1 expression. Expression data was provided through The Cancer Genome Atlas (GDC TCGA). Low/high DDR1 expression was categorized as <50% vs. ≥50% (group50).

A

	ctrl	KO2	KO6
**DDR1/GAPDH**	1	0,193	0,309

B tDDR1

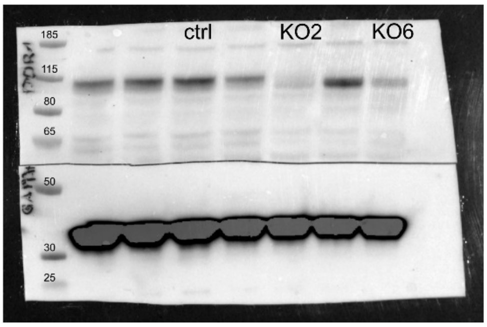

C GAPDH

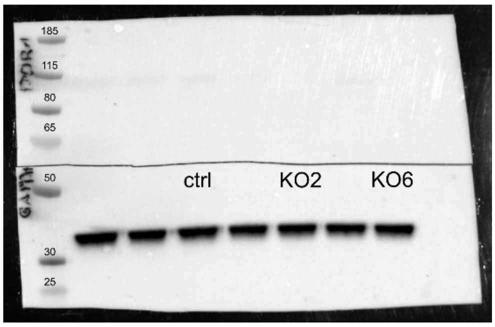

Figure S6. Intensity ratios and western blots for Supp. Fig. 2A. (**A**) Values indicate intensity ratio DDR1/GAPDH. (**B**) tDDR1 (125 kDa) and (**C**) GAPDH (37 kDa) western blot gels.
